# Modulation of respiratory dendritic cells during Klebsiella pneumonia infection

**DOI:** 10.1186/1465-9921-14-91

**Published:** 2013-09-17

**Authors:** Holger Hackstein, Sabine Kranz, Anne Lippitsch, Andreas Wachtendorf, Olivia Kershaw, Achim D Gruber, Gabriela Michel, Jürgen Lohmeyer, Gregor Bein, Nelli Baal, Susanne Herold

**Affiliations:** 1 Institute for Clinical Immunology and Transfusion Medicine, Justus-Liebig-University Giessen, Member of the German Center for Lung Research (DZL), Langhansstr. 7, D-35392, Giessen, Germany; 2 Department of Veterinary Pathology, Freie Universität Berlin, Robert-von-Ostertag-Str. 15, 14163, Berlin, Germany; 3 Department of Internal Medicine II, Klinikstrasse 33, Berlin, Germany

**Keywords:** Klebsiella pneumonia, Pneumonia, Plasmacytoid dendritic cells

## Abstract

**Background:**

Klebsiella pneumoniae is a leading cause of severe hospital-acquired respiratory tract infections and death but little is known regarding the modulation of respiratory dendritic cell (DC) subsets. Plasmacytoid DC (pDC) are specialized type 1 interferon producing cells and considered to be classical mediators of antiviral immunity.

**Method:**

By using multiparameter flow cytometry analysis we have analysed the modulation of respiratory DC subsets after intratracheal Klebsiella pneumonia infection.

**Results:**

Data indicate that pDCs and MoDC were markedly elevated in the post acute pneumonia phase when compared to mock-infected controls. Analysis of draining mediastinal lymph nodes revealed a rapid increase of activated CD103^+^ DC, CD11b^+^ DC and MoDC within 48 h post infection. Lung pDC identification during bacterial pneumonia was confirmed by extended phenotyping for 120G8, mPDCA-1 and Siglec-H expression and by demonstration of high Interferon-alpha producing capacity after cell sorting. Cytokine expression analysis of *ex vivo*-sorted respiratory DC subpopulations from infected animals revealed elevated Interferon-alpha in pDC, elevated IFN-gamma, IL-4 and IL-13 in CD103^+^ DC and IL-19 and IL-12p35 in CD11b^**+**^ DC subsets in comparison to CD11c^+^ MHC-class II^low^ cells indicating distinct functional roles. Antigen-specific naive CD4^+^ T cell stimulatory capacity of purified respiratory DC subsets was analysed in a model system with purified ovalbumin T cell receptor transgenic naive CD4^+^ responder T cells and respiratory DC subsets, pulsed with ovalbumin and matured with Klebsiella pneumoniae lysate. CD103^+^ DC and CD11b^+^ DC subsets represented the most potent naive CD4^+^ T helper cell activators.

**Conclusion:**

These results provide novel insight into the activation of respiratory DC subsets during Klebsiella pneumonia infection. The detection of increased respiratory pDC numbers in bacterial pneumonia may indicate possible novel pDC functions with respect to lung repair and regeneration.

## Introduction

Klebsiella spp. are gram-negative bacteria and important opportunistic pathogens causing life-threatening nosocomial infections [1,2]. Typical clinical presentations of Klebsiella pneumonia are nosocomial respiratory tract infections, urinary tract infections, infections of the bloodstream and premature infant intensive care unit infections [1,3-6]. Multidrug resistant Klebsiella pneumonia strains are becoming an increasingly relevant medical problem worldwide with limited clinical treatment options [7-11]. Therefore, there is a medical need for the development of novel therapeutic strategies in addition to classical antibiotic therapies. However, despite the clinical significance of Klebsiella pneumonia, little is known regarding the modulation of respiratory dendritic cells (DC) subsets during lower respiratory tract infection with Klebsiella spp.

DC are central instigators of innate and adaptive immunity regulating both inflammation and activation of antigen-specific lymphocytes [12-14]. Based on the expression of different surface markers respiratory DC can be separated in at least four subsets, plasmacytoid DC (pDC), CD103^+^ DC, CD11b^hi^ DC and monocytic DC (MoDc) [15-19]. PDC represent the most potent producers of Interferon-alpha and therefore play critical roles in antiviral immunity [20-23]. Additionally, it has been reported that pDC sense skin injury and promote wound healing through type I interferon suggesting a novel role for these professional antigen presenting cells [24].

Respiratory CD103^+^ DC have been reported to be involved in the development of airway hyperresponsiveness and to play important roles in naive CD8^+^ T cell activation and antiviral respiratory immunity [25,26]. With respect to bacterial respiratory tract infections, CD103^+^ DC have been shown to play a key role for α-Galactosylceramide-mediated protection against lethal Streptococcus pneumonia infection [27]. Together with CD11b^hi^ DC and MoDC, CD103^+^ DC have been suggested to represent the major migratory DC subsets promoting naive CD4^+^ and CD8^+^ T cell activation in the draining lymph nodes [15,26,28-30].

We have used multicolor flow cytometry to dissect and quantitate respiratory DC subsets during murine Klebsiella pneumonia. Additionally, respiratory DC subsets were purified by cell sorting from Klebsiella-infected animals and analysed for cytokine mRNA expression. The functional CD4^+^ naive T cell stimulatory capacity of purified respiratory DC subsets was determined *in vitro* after Klebsiella pneumonia lysate stimulation of ovalbumin-pulsed DCs and ovalbumin (OVA) T-cell receptor (TCR)-transgenic naive CD4^+^ responder T cells.

## Materials and methods

### Mice and treatment protocol

Specific-pathogen-free C57BL/6 (C57BL/6NCrl) and OVA TCR transgenic C57BL/6-Tg(TcraTcrb)425Cbn/J mice (18-21 g) were purchased from Charles River, Sulzfeld, Germany and maintained under specific-pathogen-free conditions. The transgenic mice express the TCR that pairs with the CD4 coreceptor and is specific for chicken OVA 323–339 in the context of MHC-class-II (I-A^b^). Wild-type mice were infected intratracheally with Klebsiella pneumonia serotype 2 (1 × 10^4^ in sterile PBS in 50 μl) as described [31] and analysed at the indicated timepoints. Mock-infected controls were treated identical but received sterile NaCl instead of Klebsiella pneumonia. Analyses were performed after approval of the regional authority board City of Giessen (#71/2009 and # A25/2009).

### Lung and mediastinal lymph node preparation

Lung single cell suspension were prepared after enzymatic digestion as described in detail elsewhere [16,17]. Absolute respiratory cell counts were enumerated with the trucount method (BD Biosciences, Germany) as described [16,17]. Trucount tubes contain a known number of fluorescent beads allowing the flow cytometer software to calculate absolute cell counts. Single cell suspension from mediastinal lymph nodes (MLN) were minced and digested in RPMI 1640/10% FCS (PAA laboratories, Germany) with 10 U/ml DNase and 1 mg/ml Collagenase A (Roche, Germany) for 30 min at 37 C, resuspended with a 20 G 1 ½ canule (0.9 × 40 mm, BD, Germany), mashed through a 70 μm cell strainer and washed two times with HBSS (7 min, 400 g; PAA laboratories, Germany).

### Flow cytometry and fluorescent activated cell sorting

Cellular phenotyping was performed on a BD CantoII flow cytometer and fluorescent activated cell sorting was performed on a BD ARIA3 cell sorter (Becton Dickinson, San Jose, CA, USA). The following fluorochrome-labelled monoclonal antibodies conjugated to FITC, PE, PeCy7, PerCPCy5.5, APC, APC-Cy7, Pacific Blue and appropriate isotype controls were used for surface staining according to the manufacturer’s instructions: CD11b, CD11c, CD45, CD64, CD86, CD103, CD274, I-A^b^, GR-1, Ly-6G, F4/80, NK1.1, FcϵRIα (MAR-1), Siglec-H, Siglec-F (BD Biosciences, Germany), mPDCA-1 (MiltenyiBiotec, Germany), 120G8 (Dendritics, France). Surface mAb or isotype staining time was 30 min on ice and cells were washed with staining buffer (1 × HBSS, PAA, Germany) at 400 g, 5 min, room temperature (RT) before analysis. The number of acquired events was ≥ 500,000 after surface stainings.

Highly purified naive OVA TCR transgenic CD4+ T cells were prepared from spleen cell suspensions. First, MHC-class-II and CD19 positive cells were depleted with magnetic-beads (MiltenyiBiotec) on an Auto-MACS magnetic-bead sorter (MiltenyiBiotec) and subsequently naive CD4+ T cells were sorted on the ARIA3 sorter after surface staining with a lineage cocktail (CD8a, CD25, CD11b, CD11c, CD45R, CD49b) and CD62L mAbs to identify naive T cells . The post sort purity of CD4^+^ CD62L^+^CD44^dim^ cells was >98%. Respiratory DC subsets were sorted on the ARIA3 cell sorter (purity >98%; Additional file 1: Figure S1). Viability of cells was > 90% as indicated by sytox blue (Life Technologies, Germany) staining. All mabs were ordered from Biolegend, Germany unless indicated otherwise.

### Gating strategy for respiratory leukocyte subset discrimination

The gating strategy for the respiratory subsets, including respiratory DC subsets has been described recently in detail [17,19]. Briefly, respiratory leukocytes were identified by CD45 expression. Out of the CD45+ cells, neutrophils were identified by GR1^bright^CD11b^bright^ expression. Subsequently, out of the neutrophil negative fraction, macrophages were identified as SiglecF^++^F4/80^+^ double positive cells. Respiratory dendritic cells (DC) were identified according to CD11c^+^Siglec-F^neg^ NK1.1^neg^ expression to exclude autofluorescent macrophages and NK cells and further dissected into pDC (120 g8^+^ CD11b^neg^), CD103 DC (CD103^+^ CD11b^neg^) and CD11b DC (CD11b^+^ CD103^neg^). MoDC were discriminated from CD11b DC based on CD64 and MAR1 expression as described recently [19]. In MLN, cells were additionally stained for CCR7 to identify migrating CD103 DC, CD11b DC and MoDC (Additional file 2: Figure S2). Fluorescence minus one (FMO) isotype controls were used regularly for proper identification of marker positive subsets.

### Naive CD4+ T cell proliferation assay

Purified DC subsets (1 × 10^4^/well) were incubated in culture medium (±100 μg/ml endotoxin-free OVA; Hyglos, Germany) with carboxyfluorescein diacetate succinimidyl ester (CFSE) labeled purified naive CD4^+^ OVA TCR transgenic responder cells (1 × 10^5^/well) in 96 well round bottom wells (Greiner, Germany) for five days. Klebsiella pneumonia lysate was prepared as described [16] and added at a concentration of 10 ul/ml (1:100) 4 h after OVA for DC maturation. DC maturation with different Klebsiella pneumonia lysate dilutions (1:10–1:500) was controlled by flow cytometry analysis of CD86 and MHC-class II expression on respiratory DC (Additional file 3: Figure S3). Culture medium consisted of RPMI 1640 with L-glutamine (PAA Laboratories), penicillin/streptomycin (PAN Biotech), sodium-pyruvate (Gibco), nonessential aminoacids (Sigma), Hepes buffer (Gibco), and 10% heat inactivated FCS (PAA Laboratories). CFSE labeling concentration was 1 μM and was performed according to the manufacturer instructions (Vybrant CFDA-Cell Tracer kit, Molecular probes, Eugene, USA). Positive controls consisted of *in vitro* bone-marrow-derived DC (expanded as described [32]) with OVA and responder cells. Negative controls consisted of responder cells cultured with OVA in the absence of stimulator cells and responder cells cultured with stimulator cells in the absence of ovalbumin. Proliferation was quantitated by measurement of CFSE-dilution in responder cells by flow cytometry as described [33]. Positive bone-marrow-derived control DC pulsed with OVA induced naive CD4+ responder proliferation rates of >80%. Proliferation rates in negative controls were < 5%.

### IFN-α induction and quantitation

IFN-α production was stimulated with CpG ODN2216 (6 μg/ml) in sorted cell subsets after overnight culture in RPMI 1640 with 10% FCS (PAA Laboratories). IFN-α was quantitated in cellular supernatants on the FACS CantoII flow cytometer in parallel to recombinant cytokine standards by using the cytometric bead assay technology according to the manufacturer instructions (Flowcytomix, eBioscience, Germany).

### Cytokine mRNA quantification

Cytokine mRNA expression was determined in sorted respiratory DC subsets from Klebsiella-infected animals by quantitative RT-PCR on an ABI TaqMan Step-One plus real time PCR system (Applied Biosystems, Germany) with the RT^**2**^ profiler PCR assay (Qiagen, Germany). RNA was isolated with RNeasy Mini Kit (Qiagen) and transcribed into cDNA with the RT^2^ Preamp cDNA Synthesis Kit (Qiagen) according to the manufacturer instructions.

### Statistical analyses

The significance of differences between groups were analysed by one-way ANOVA and Tukey post-test for multiple comparisons. A p-value < 0.05 was considered significant. Statistical analyses were performed with Prism 5.02 software (Graphpad software, Inc., USA).

## Results

### Respiratory dendritic cell subsets and Klebsiella pneumonia infection

**Figure 1:**
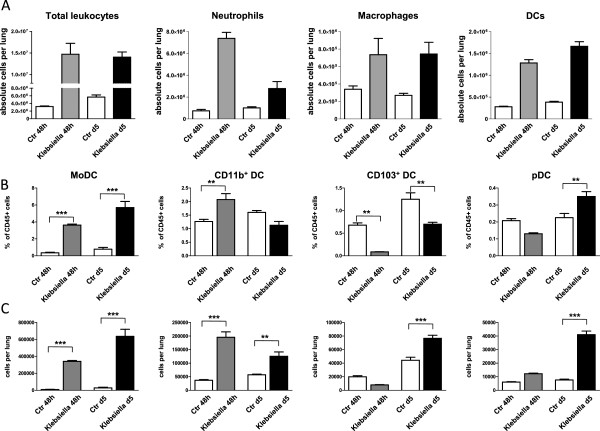
**Dissection of respiratory leukocyte and DC subsets (MoDC, CD11b**^**hi **^**DC, CD103**^**+ **^**DC, pDC) during Klebsiella pneumonia infection.** Respiratory leukocyte subsets were dissected by flow cytometry as described in Materials and methods **(A-C)**. Relative frequencies and absolute numbers of respiratory DC subsets were quantitated at indicated timepoints and compared to mock-infected control animals **(B, C)**. Mean ± SEM; n ≥ 4; *p < 0.05; **p < 0.01; ***p < 0.001 versus mock-infected controls.

**Figure 2:**
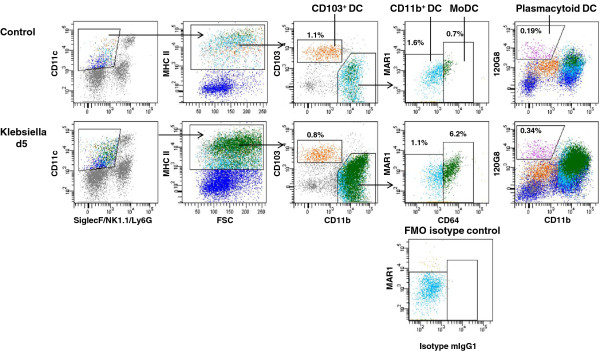
**Respiratory DC subsets during Klebsiella pneumonia infection.** Dotplot staining and gating of respiratory DC subsets (CD103^+^ DC, CD11b^+^ DC, MoDC, pDC) in Klebsiella-infected animals and mock-infected controls. Fluorescence minus one (FMO) isotype control for proper identification of CD64^+^ MoDC.

**Figure 3:**
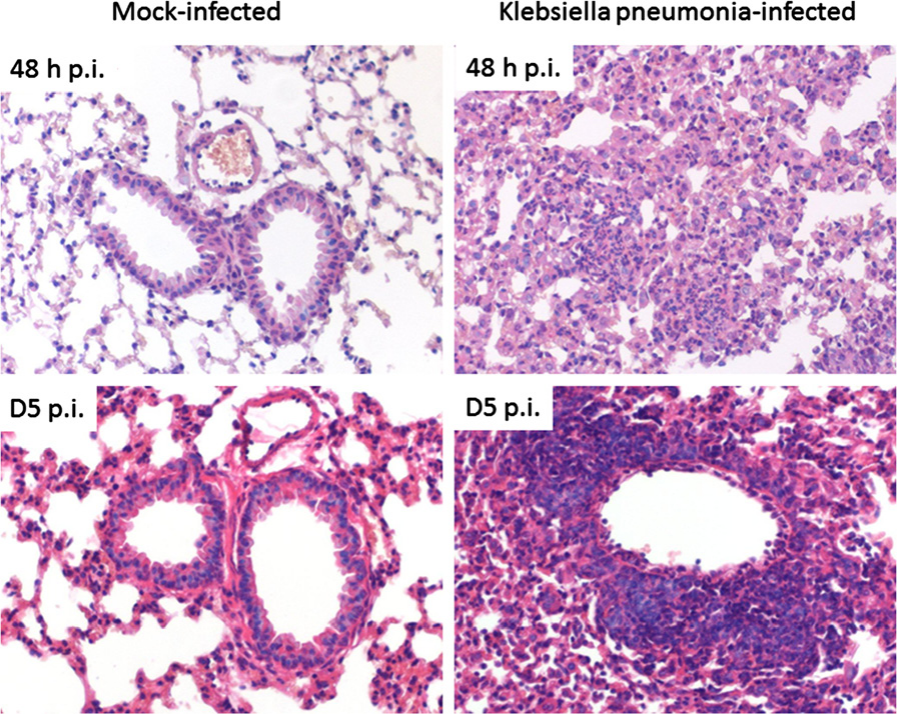
**Significant lung pathology in Klebsiella pneumonia infected animals.** Histopathology of mouse lungs two (upper row) and five days (bottom row) after mock (left column) and K. pneumoniae (right column) infection: No changes were present in mock infected mice. In contrast, extensive interstitial infiltration by predominantly neutrophils and macrophages was present at two days after infection of mice with K. pneumoniae. After five days, additional, predominantly perivascular, lymphocytic infiltrates were seen. Hematoxylin & Eosin stained lung sections at 200 × magnification.

By using multiparameter flow cytometry we analysed absolute and relative changes of major respiratory leukocyte populations including neutrophils, macrophages and DC during acute (48 h p.i.) and post acute (d5 p.i.) respiratory Klebsiella pneumonia infection (Figure 1A). Total DC were further dissected by flow cytometry into four major subsets, MoDC, CD11b^+^ DC, CD103^**+**^ DC and pDC and relative DC subset frequencies and absolute DC subset numbers were calculated (Figure 1B,C; Figure 2). Results indicated that total respiratory DCs in general were increased both at 48 h p.i and d5 p.i. but the dissection of DC subsets revealed different patterns for each subpopulation (Figure 1B,C). Early after infection (48 h p.i.), relative CD103^+^ DC numbers were significantly decreased in accordance with their reported function to rapidly transport antigen to regional lymph nodes [34]. Unexpectedly, with respect to pDC representing the rarest subset, we found a marked relative and absolute elevation of respiratory pDC numbers in the post acute phase of Klebsiella pneumonia (d5 p.i.) when compared to mock-infected controls (p < 0.01; Figure 1B,C). Additionally, absolute numbers of respiratory CD11b^+^ DC and MoDC were markedly increased at 48 h and day 5 p.i. (Figure 1C). The massive leukocyte subset changes detected by flow cytometry correlated well with histopathology exhibiting extensive cellular infiltrates and cellular necrosis indicating severe bacterial pneumonia (Figure 3).

### Confirmation of functional respiratory pDC during Klebsiella pneumonia infection by extended phenotyping and cell sorting

**Figure 4:**
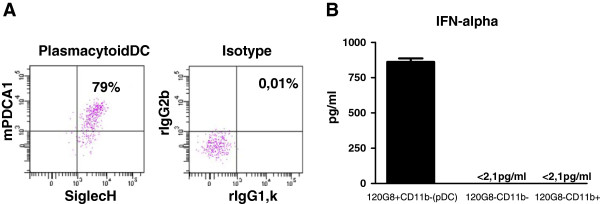
**Confirmation of respiratory pDC identity during Klebsiella infection (d5 p.i.) by extended phenotyping and cell sorting.** Identity of pDC during Klebsiella pneumonia was confirmed by additional phenotyping of CD11c + 120 g8+ CD11b^neg^ cells for mPDCA-1 and Siglec-H in parallel with isotype controls **(A)**. Functional ability of respiratory pDC from Klebsiella infected animals to produce IFN-alpha was confirmed by cell sorting and CPG ODN stimulation in comparison to control populations **(B)**. Respiratory pDC during Klebsiella pneumonia are CD11b^**neg**^ 120G8^+^ Siglec-H^+^ mPDCA-1^+^ cells **(A, B)** and exhibit the capacity to produce large amounts of IFN-α **(B)**. Mean ± SEM; n ≥ 3.

Given the fact, that Klebsielle pneumonia infection induced major respiratory inflammation we discussed the possibility that pDC identification by flow cytometry might have been confounded by unspecific surface marker upregulation due to the inflammatory process. Accordingly, we extended pDC surface marker analysis on Klebsiella-infected animals (d5 p.i.) and included Siglec-H and mPDCA-1 mAb surface staining (Figure 4A). These experiments confirmed expression of Siglec-H and mPDCA-1 on respiratory pDC during Klebsiella pneumonia. Additionally, in order to use an independent functional method we sorted lung 120G8^+^ CD11b^-^ CD11c^+^ CD45^+^ putative respiratory pDC (d5 p.i.) and tested their capacity to produce IFN-α. These experiments confirmed high IFN-α producing capacity in respiratory pDC from Klebsiella-infected animals in comparison to non-pDC control fractions (Figure 4B). These data demonstrated that the immunophenotypically identified pDC during bacterial pneumonia, represented viable and functional respiratory pDC.

### Distinct cytokine expression of purified respiratory DC subsets during Klebsiella pneumonia infection

**Figure 5:**
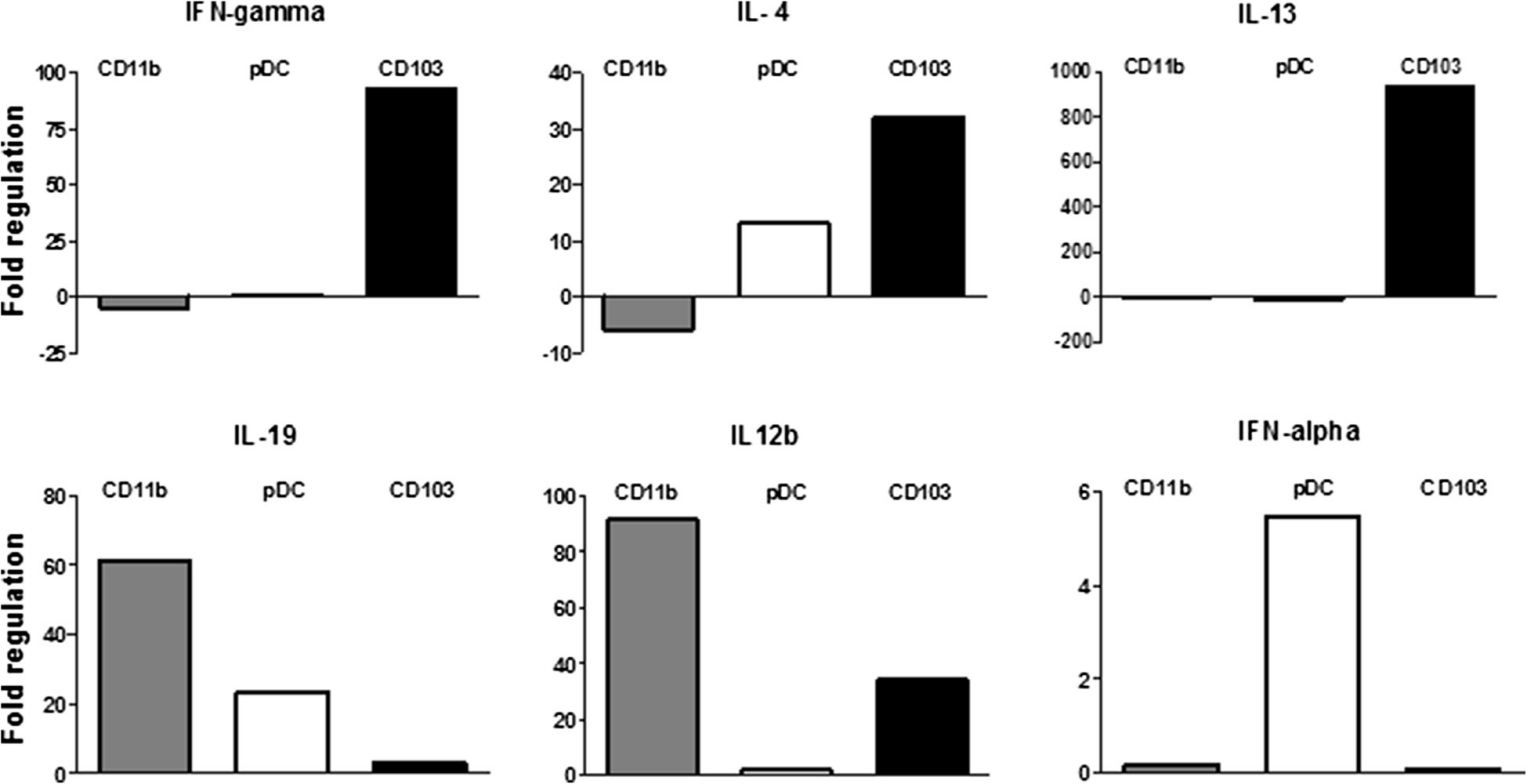
**Relative mRNA cytokine expression of respiratory CD11b**^**+ **^**DC, CD103**^**+ **^**DC, pDC from Klebsiella pneumoniae-infected animals.** Semi-quantitative mRNA cytokine expression was analysed by real time RT-PCR in sorted respiratory DC subsets from d5-infected animals. Mean fold regulation in relation to MHC-II^low^ CD11c^+^ cells; n ≥ 3.

To determine the cytokine expression of respiratory DC subsets during Klebsiella pneumonia we sorted pDC, CD103^+^ DC, CD11b^+^ DC and CD11c^+^ MHC-class II^low^ cells from Klebsiella-infected animals (d5 p.i.) to high purity (>98%) and analysed mRNA expression by quantitative real time RT-PCR. Controls included genomic DNA controls, reverse transcriptase controls and positive PCR controls. Relative cytokine expression of pDC, CD103^+^ DC and CD11b^+^ DC in comparison to CD11c^+^ MHC-class II^low^ cells was calculated by the 2^(−ΔΔ CT)^ method to indicate differences between the subsets (Figure 5). CD11c^+^ MHC-class II^low^ cells were selected as common reference because we wanted to display relative cytokine expression differences in-between pDC, CD103^+^ DC and CD11b^+^ DC. Respiratory pDC exhibited elevated IFN-α expression indicating activation during bacterial pneumonia. In contrast, CD103^+^ DC exhibited increased IFN-γ, IL-4 and IL-13 expression, whereas CD11b DC showed elevated expression of IL-12p35 and IL-19 in comparison to CD11c^+^ MHC-class II^low^ cells.

### Different modulation of MHC class II, CD86 and CD274 expression on respiratory DC subsets during acute Klebsiella pneumonia infection

**Figure 6:**
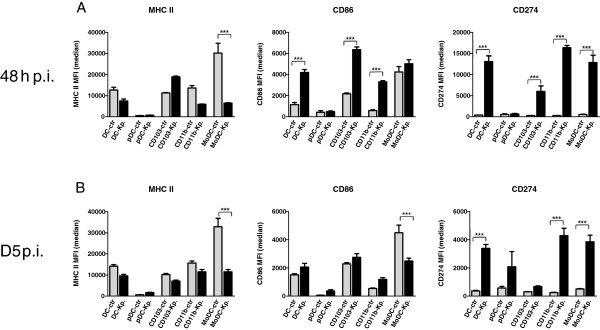
**Respiratory DC-subset specific expression of MHC-class II, CD86 and CD274 at different timepoints during acute Klebsiella pneumonia infection.** Surface MHC-class II, CD86 and CD274 on total respiratory DC (DC) and DC subsets (pDC, CD103^+^ DC, MoDC, CD11b^+^ DC) were analyzed by flow cytometry during acute and post-acute Klebsiella pneumonia (Kp) infection (48 h and d5 p.i.) and compared to mock-infected control (ctr) animals **(A, B)**. MFI is median fluorescence intensity; mean ± SEM; n ≥ 3; *p < 0.05; **p < 0.01; ***p < 0.001 versus mock-infected controls **(A-B)**.

Expression of MHC-class II, CD86 and CD274 represent functionally important molecules of antigen presenting cells to regulate CD4^+^ T responder cell activation. Accordingly, we quantitated MHC-class II, CD86 and CD274 surface expression on total respiratory DC and DC subsets to determine the modulation during acute and post-acute respiratory Klebsiella pneumonia infection (Figure 6A,B). Results demonstrated that at 48 h p.i. and at d5 p.i. respiratory MoDC and to a lesser extent CD11b^+^ DC from Klebsiella pneumonia-infected animals exhibited reduced MHC-class II expression. In contrast, costimulatory CD86 expression was markedly increased at 48 h p.i. on respiratory CD103^+^ DC and CD11b^+^ DC of infected animals when compared to mock-infected-controls. pDC and MoDC did not exhibit major CD86 expression changes at 48 h p.i. Moreover, MoDC exhibited at d5 p.i. reduced CD86 expression which may be related to the differentiation of newly arrived DC. The immunoregulatory molecule CD274 was most markedly induced on CD11b^+^ DC and MoDC both at 48 h p.i. and d5 p.i. whereas expression on CD103^+^ DC and pDC was less affected. These results indicated, that respiratory DC subsets exhibited major heterogeneity with respect to MHC-class II, CD86 and CD274 expression during respiratory Klebsiella infection suggesting different functional capacity to activate responder T cells. These differences would have been masked, if respiratory DC were analysed as a single CD11c^+^ Siglec F^neg^ MHC-II^+^ population without subset discrimination.

### Klebsiella pneumoniae-activated respiratory DC subsets exhibit different naive CD4^+^ T cell stimulatory capacity

**Figure 7:**
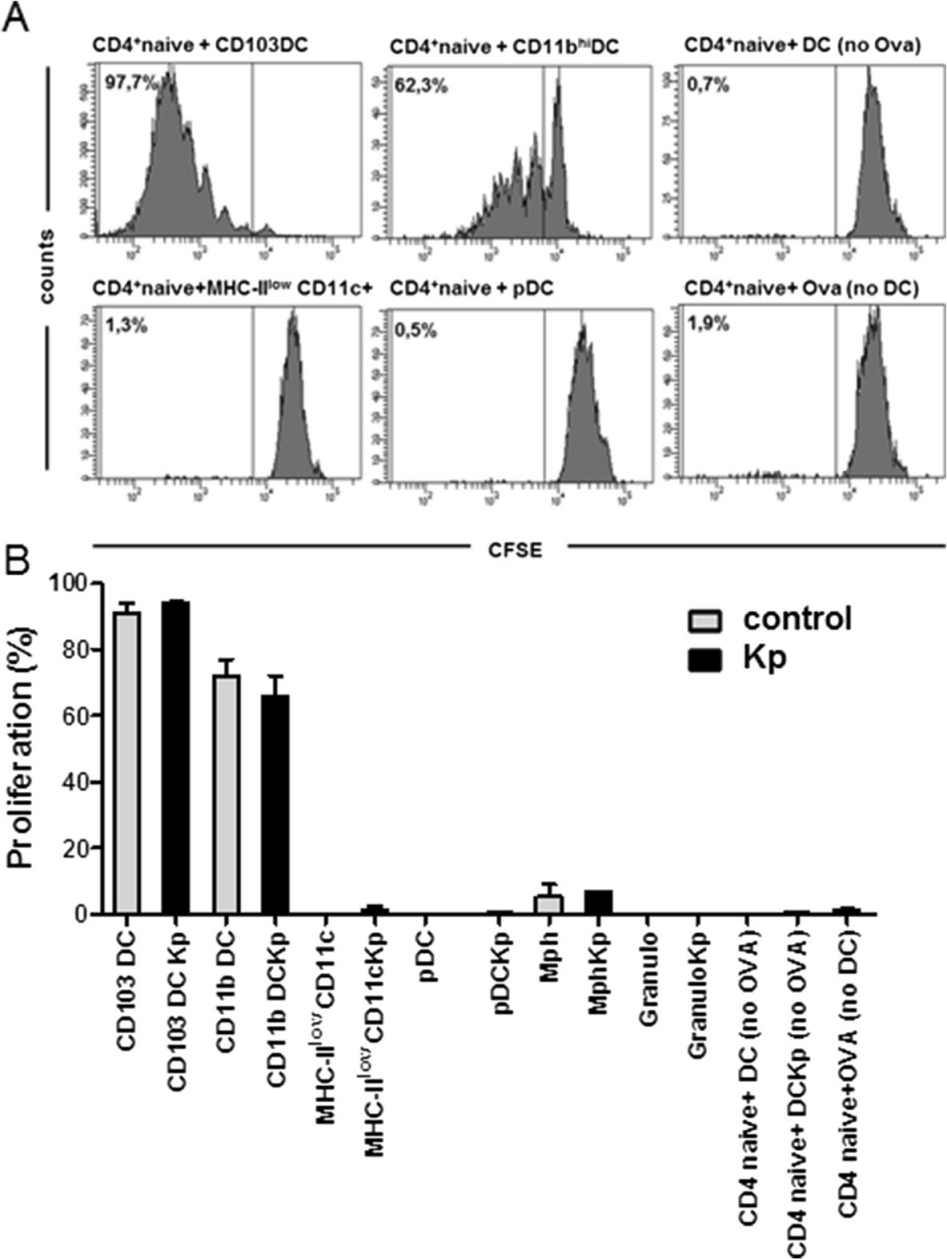
**Different capacity of purified Klebsiella pneumoniae-stimulated respiratory DC subsets to activate OVA TCR-transgenic naive CD4**^**+ **^**responder T cells in a CFSE-dilution assay.** Respiratory DC subsets were sorted to high purity (>98%), pulsed with the model antigen OVA and matured with Klebsiella pneumoniae lysate (Kp). Naïve CD4^+^ responder T cells were sorted from OVA TCR transgenic animals and labeled with CFSE. Negative controls included DC without Klebsiella lysate (control), responder T cells cultured without DC (no DC) and DC-T cell co-cultures without OVA (no OVA). Proliferation was quantitated by CFSE-dilution of responder T cells **(A,B)**. Mean ± SEM; n ≥ 3.

In order to determine the naive CD4^+^ T cell stimulatory capacity of purified respiratory DC subsets activated by Klebsiella pneumonia we used an *in vitro* model system with CFSE-labelled OVA TCR transgenic CD4^+^ T helper cells as antigen-specific responder cells. Stimulatory respiratory DC subsets were sorted to high purity (>98% for each subset) labelled with the model antigen OVA and matured with Klebsiella pneumonia lysate. Additional controls included sorted respiratory macrophages and granulocytes and *in vitro* bone-marrow-derived DC without OVA (negative control). Untouched naive CD4^+^ TCR transgenic responder cells were magnetic-bead sorted and the phenotype after sorting was controlled by surface CD4, CD62L and CD44 staining (purity >98%). Results indicated that Klebsiella pneumonia-activated CD103^+^ DC and CD11b^+^ DC subsets represented the most efficient stimulators of purified naive CD4 T responder cells (Figure 7; mean proliferation rates 95% and 64%, respectively). In contrast, Klebsiella pneumonia-activated pDC and CD11c^+^ MHC-class II^low^ cells, as well as granulocytes and macrophages induced no or only minor naive CD4 T cell proliferation (< 5%). Interestingly, sorted DC subsets that were not activated with Klebsiella pneumonia induced comparable CD4^+^ T cell proliferation indicating that Klebsiella may induce both activatory and inhibitory effects on DC. Control experiments with cultures of DC + CD4^+^ T cells without OVA and CD4^+^ T cells + OVA without DC showed no responder cell proliferation and confirmed the antigen-specificity of the assay (Figure 7A,B). Naïve CD4^+^ T cell stimulatory capacity of purified, Klebsiella activated respiratory DC subsets correlated closely with MHC-class II and CD86 expression of respiratory DC subsets from Klebsiella-infected animals (Figure 7) underlining the functional importance of MHC-class II and CD86 expression. Overall, these results indicated marked differences of Klebsiella-activated respiratory DC subsets to activate naïve CD4^+^ T cells.

### Rapid accumulation of activated CD103^+^ DC, CD11b^+^ DC and MoDC in the MLN

**Figure 8:**
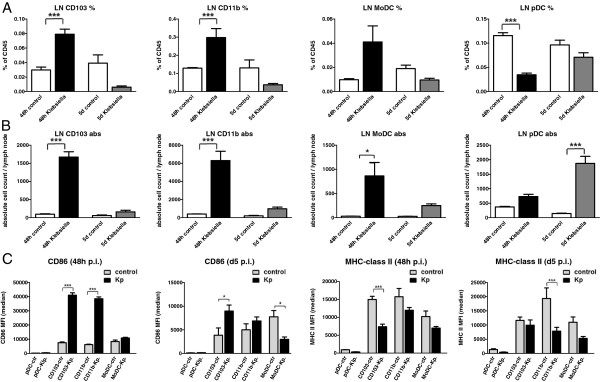
**Quantitative and phenotypic analysis of DC subsets in the MLN at different time points during Klebsiella pneumonia infection.** Relative and absolute CD103^+^ DC, CD11b^+^ DC, MoDC and pDC subset numbers **(A, B)** and phenotypic markers **(C)** were analyzed at indicated time points in the MLN and compared to mock-infected controls by flow cytometry as described in Materials and methods. Mean ± SEM; n ≥ 3; *p < 0.05; **p < 0.01; ***p < 0.001 versus mock-infected controls.

Analysis of DC subset changes in the draining MLN revealed a marked increase of relative and absolute CCR7^+^ CD103^+^DC, CD11b^+^ DC and MoDC numbers within 48 h p.i. whereas absolute pDC numbers were elevated on D5 p.i. (Figure 8A,B). Phenotypic analysis of activatory CD86 expression indicated significant upregulation on CD103^+^ DC and CD11b^+^ DC in contrast to pDC and MoDC (Figure 8 C). Interestingly, MHC-class II expression was impaired to different extent on all DC subsets in comparison to mock-infected controls again suggesting that Klebsiella pneumonia infection may induce both activatory and inhibitory effects on antigen presenting cells.

## Discussion

Klebsiella pneumoniae is a frequent multidrug-resistant emerging pathogen playing an increasingly important role in severe nosocomial infections [7,8,10,11]. DC are central regulators of innate and adaptive immunity against different kinds of pathogens [35-37]. Based on different developmental pathways, surface markers and tissue localizations, DC can be divided into distinct subsets exhibiting specific functions [38]. By using multiparameter flow cytometry and cell sorting we have determined the number and activation of respiratory DC subsets during experimental Klebsiella pneumoniae infection.

Subset-specific respiratory DC quantification of Klebsiella-infected animals showed that the rare pDC population were markedly elevated in the post acute disease phase. We confirmed the identity of functional respiratory pDC during bacterial pneumonia by extended phenotyping and demonstration of large IFN-alpha production capacity after cell sorting. Moreover, elevated IFN-alpha mRNA expression was demonstrated in purified respiratory pDC sorted from Klebsiella-infected animals. Unexpectedly, cytokine mRNA analysis indicated IL-4 and IL-13 expression in CD103 DC and IL-19 expression in CD11b DC. Due to the rather low cytokine mRNA expression, marked pulmonary inflammation and the technical possibility of contaminating innate lymphoid cells, further detailed studies using combinations of different methods are required to dissect the cytokine producing capacity of respiratory DC subsets during gramnegative bacterial pneumonia.

The accumulation of functional respiratory pDC during bacterial pneumonia has to the best of our knowledge not been reported so far and raises the question regarding potential novel functions of pDC in bacterial pneumonia. Respiratory pDC may play a role in tissue repair. Gregorio et al. recently reported, that pDC accumulate in large numbers in injured skin areas and express IFN-α [24]. The expression of IFN-α was shown to be related to the detection of self nucleic-acids that were released due to the skin injury. Additionally, it was demonstrated that pDC depletion significantly delayed wound healing suggesting a functional role for these rare DC subset in tissue repair. Alternatively, accumulation of pDC in the post acute bacterial pneumonia phase may be related to immunoregulatory functions of these cells. PDC have the capacity to control both CD4+ and CD8+ T cell activation [39]. Antigen presentation to CD4 T cells via pDC has been described to inhibit T helper 1 and T helper 17 immune responses, suggesting a possible role to prevent T helper-dependent automimmunity [40]. In a different study using a conditional plasmacytoid depletion model, pDC were demonstrated to play an important role in the stimulation of antiviral CD8^+^ T cell responses [41].

Our results indicate that Klebsiella pneumonia may exhibit additionally inhibitory effects on DC. The *ex vivo* analysis of MHC-class II expression on lung DC subsets of Klebsiella pneumonia infected animals consistently indicated decreased MHC-class II expression in comparison to mock-infected controls. This may be related to the recruitement or differentiation of newly arrived DC. However, analysis of CCR7 migrating DC in the draining MLN during infection additionally indicated decreased MHC-class II expression suggesting Klebsiella pneumonia-mediated suppression. Additionally, analysis of the inhibitory molecule CD274 on lung DC during Klebsiella infection revealed a massive upregulation on CD11b DC and MoDC and to a lesser extent on CD103 DC suggesting another pathway of Klebsiella pneumonia-mediated suppression. Klebsiella-mediated induction of inhibitory molecule CD274 on lung DC may be important in limiting antibacterial effector T cell responses and promoting infection-associated immunosuppression [42,43]. In this context it would be interesting to establish technologies allowing precise DC subset specific enumeration of bacterial load. Sorting strategies are limited due to a number of technical factors, such as passive bacteria attachment, complex DC phenotype, low DC frequency. Therefore, development of other technologies, such as combination of specific staining of bacterial-related gene products with DC subset markers might be more successful [44].

Our results using ovalbumin-pulsed, Klebsiella pneumoniae-stimulated purified pDC as stimulators of TCR transgenic naive CD4^+^ T cells confirmed the finding, that pDC are not efficient CD4^+^ T cell stimulators. In direct comparison to other respiratory DC subsets, Klebsiella pneumonia-stimulated CD103^+^ DC and CD11b DC represented the most efficient naïve CD4^+^ T cell activators. Moreover, MHC-class II and CD86 surface expression, representing important functional molecules required for MHC-class II restricted CD4 activation were markedly reduced on respiratory pDC from Klebsiella-infected animals compared to either CD103^+^ DC and CD11b DC. Additionally, analysis of draining MLN during infection revealed rapid relative and absolute accumulation of CD103 DC, CD11b DC and MoDC numbers within 48 h p.i. whereas pDC numbers did exhibit only an absolute increase at d5 p.i. indicating a different role for T cell activation.

In summary, the present study has revealed marked quantitative and qualitative differences for respiratory DC subsets during sublethal respiratory Klebsiella pneumonia infection. Both the *in vivo* as well as the *in vitro* data underline the importance of differentiating respiratory DC subsets during Klebsiella pneumonia. The analysis of lung DC as a single cell population during infection would have masked many of these differences. Our study highlights that these lung subsets exhibit different migration kinetics, different activatory/regulatory surface molecule expression and different functional CD4 activatory capacity. With respect to the novel finding of increased pDC during Klebsiella infection, future studies are necessary to address the functional role of pDC during bacterial pneumonia. The use of *in vivo* DC subset depletion models in future studies will provide additional insight into the relevance of DC subsets for CD4 and CD8 T effector cell expansion during Klebsiella pneumonia infection.

## Competing interests

The authors declare that they have no competing interests.

## Authors’ contributions

HH, NB, JL, GB and SH designed the study. HH drafted the manuscript and performed the statistical analysis. NB, AW and SK performed animal experiments. GM, AL and NB performed *in vitro* immunoassays and expression analysis. OK and AG performed the histopathological analysis. All authors read and approve the final manuscript.

## Supplementary Material

Additional file 1: Figure S1 Gating strategy and post-sort controls for pDC, MHC-II^Iow^ CD11c^+^ cells, CD103 DC and CD11b DC. Dotplot staining of sorting gates (A) and post-sort controls with % cell purity (B) for pDC, MHC-II^Iow^ CD11c^+^ cells, CD103 DC and CD11b DC. Click here for file

Additional file 2: Figure S2 Dissection of DC subsets in the MLN during Klebsiella pneumonia infection. Dotplot staining and gating of MLN CD103^+^ DC, CD11b^+^ DC, MoDC and pDC subsets in Klebsiella-infected animals and mock-infected controls. Migratory CD11c^+^ CD103^+^ DC, CD11b^+^ DC and MoDC were identified as MHC-class II^+^ CCR7^+^ cells and discriminated according to differential CD103, CD11b, MAR1 and CD64 expression. MLN pDC were identified as CD11c^+^ 120G8^+^ CD11b^neg^ cells. Fluorescence minus one controls (FMO) were used for proper identification of CCR7^+^ and CD64^+^ populations. Click here for file

Additional file 3: Figure S3 Maturation control for Klebsiella pneumonia lysate. Lung homogenate was stimulated with indicated concentrations of Kp lysate and median fluorescence intensity (MFI) for CD86 and MHC-II expression was quantitated after 24 h on CD11c^+^ Siglec F^neg^ NK1.1^neg^ DC by flow cytometry. Mean ± SEM; n ≥ 3. Click here for file
