# Supplementary figures and images for: Modulation of respiratory dendritic cells during Klebsiella pneumonia infection
Source: Respir Res. 2013 Sep 17;14(1):91. doi: 10.1186/1465-9921-14-91 (PMC3848864; doi:10.1186/1465-9921-14-91)

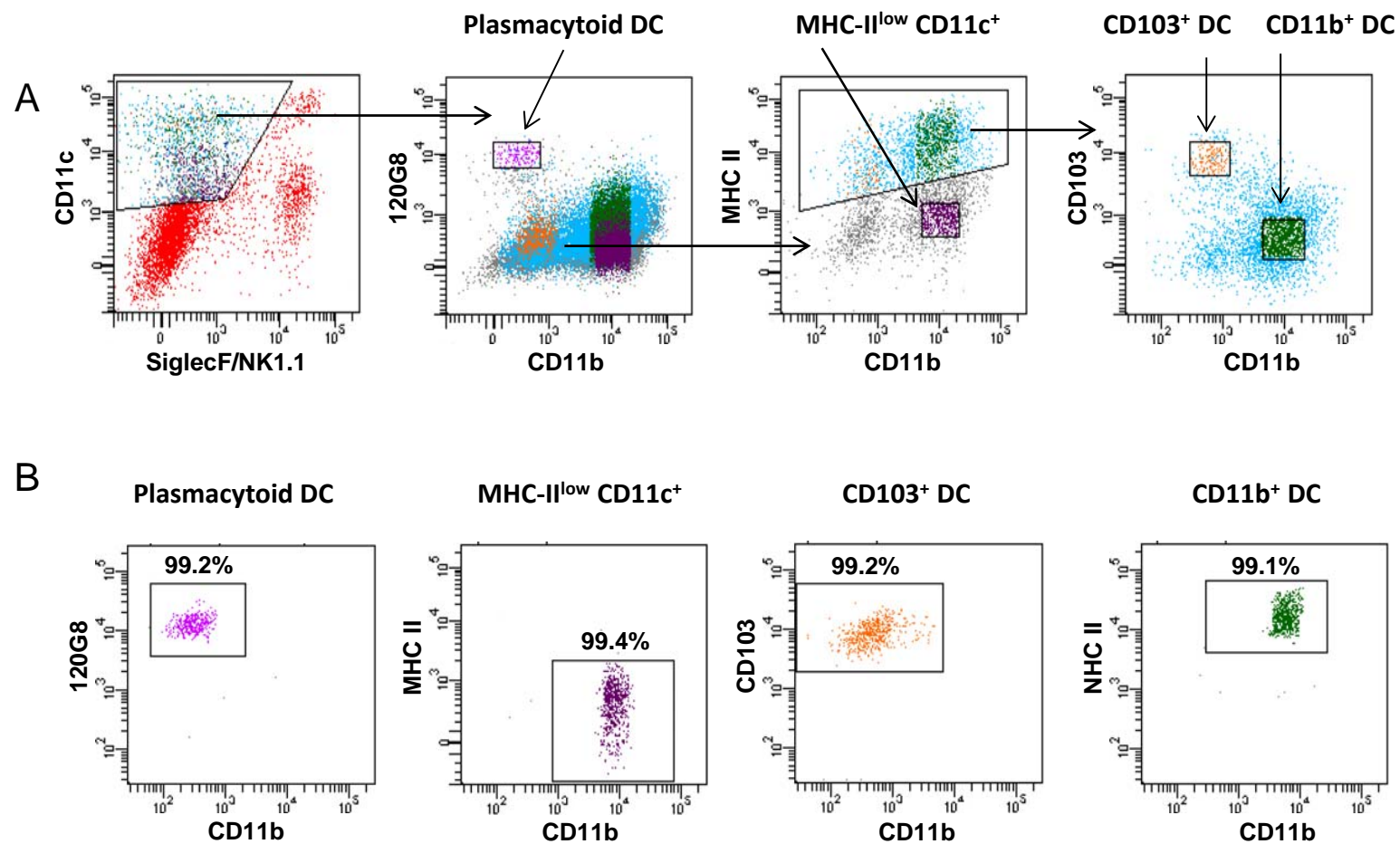

Supplementary Figure 1

Supplement: Additional file 1: Figure S1 — Gating strategy and post-sort controls for pDC, MHC-IIIow CD11c+ cells, CD103 DC and CD11b DC. Dotplot staining of sorting gates (A) and post-sort controls with % cell purity (B) for pDC, MHC-IIIow CD11c+ cells, CD103 DC and CD11b DC. [file 1465-9921-14-91-S1.pdf]

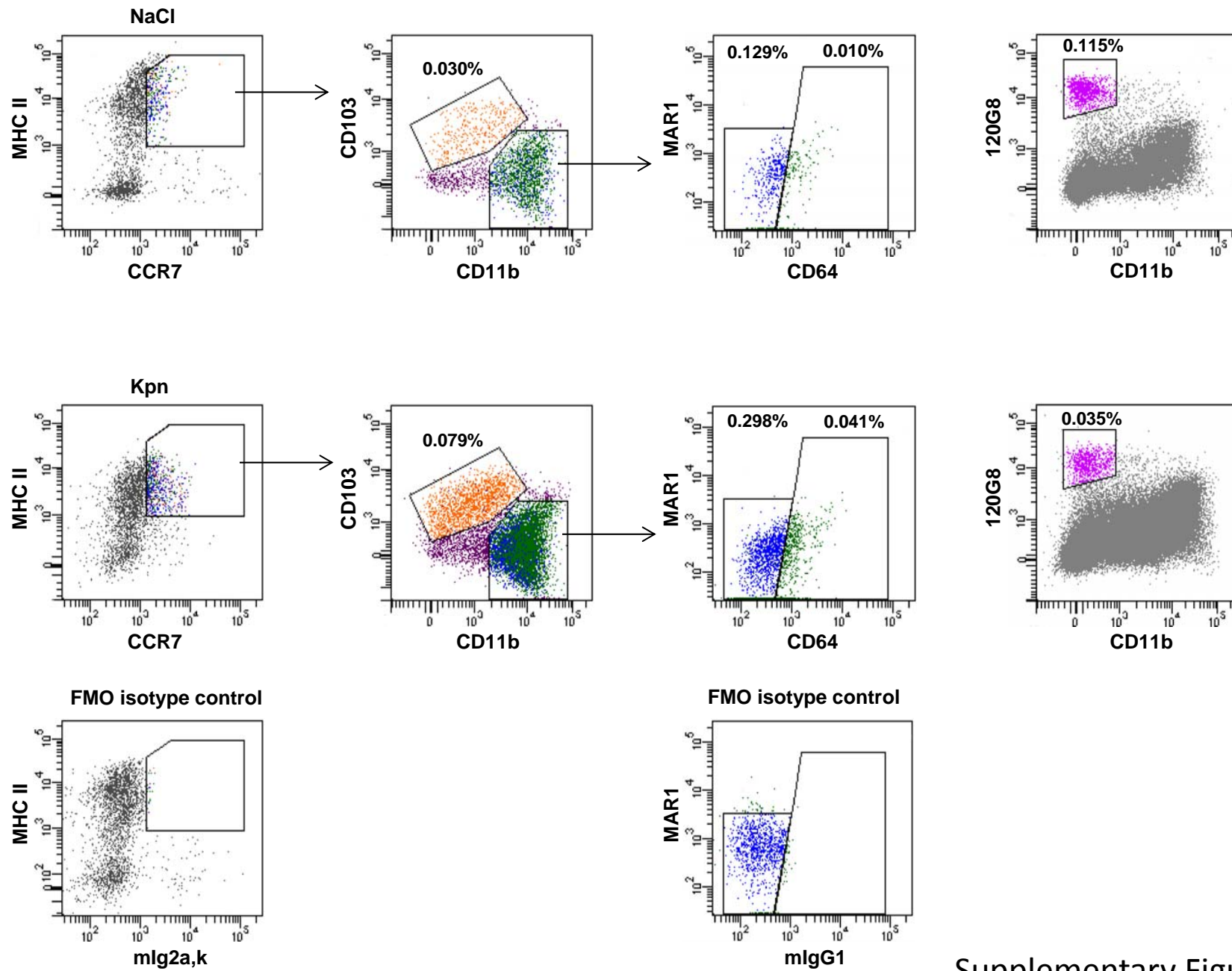

Supplementary Figure 2

Supplement: Additional file 2: Figure S2 — Dissection of DC subsets in the MLN during Klebsiella pneumonia infection. Dotplot staining and gating of MLN CD103+ DC, CD11b+ DC, MoDC and pDC subsets in Klebsiella-infected animals and mock-infected controls. Migratory CD11c+ CD103+ DC, CD11b+ DC and MoDC were identified as MHC-class II+ CCR7+ cells and discriminated according to differential CD103, CD11b, MAR1 and CD64 expression. MLN pDC were identified as CD11c+ 120G8+ CD11bneg cells. Fluorescence minus one controls (FMO) were used for proper identification of CCR7+ and CD64+ populations. [file 1465-9921-14-91-S2.pdf]

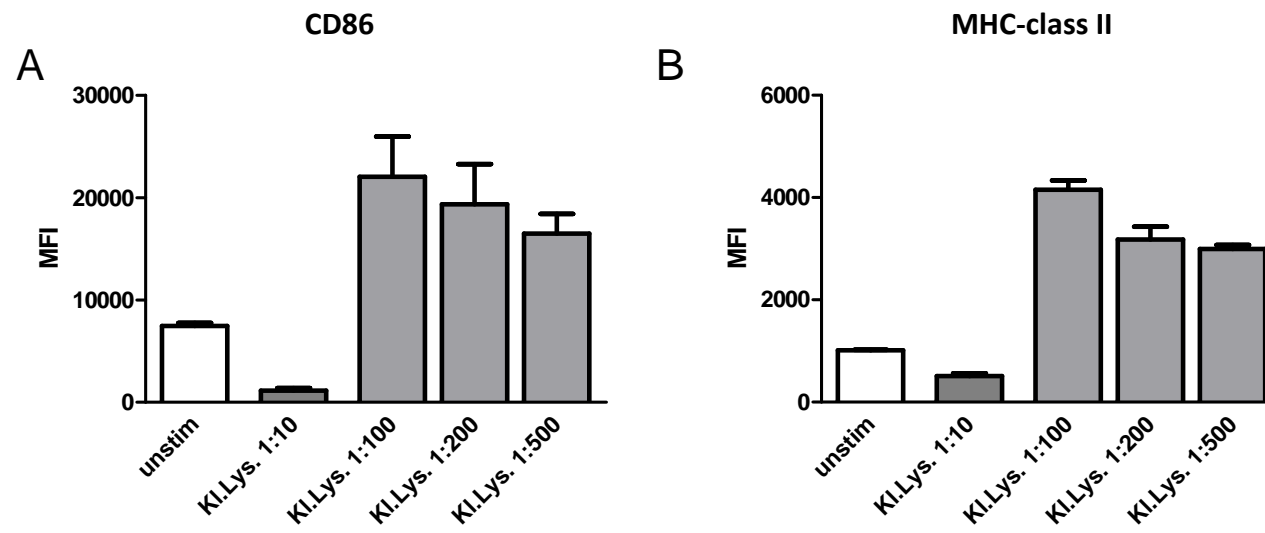

Supplementary Figure 3

Supplement: Additional file 3: Figure S3 — Maturation control for Klebsiella pneumonia lysate. Lung homogenate was stimulated with indicated concentrations of Kp lysate and median fluorescence intensity (MFI) for CD86 and MHC-II expression was quantitated after 24 h on CD11c+ Siglec Fneg NK1.1neg DC by flow cytometry. Mean ± SEM; n ≥ 3. [file 1465-9921-14-91-S3.pdf]
